# Landscape of gene fusions in epithelial cancers: seq and ye shall find

**DOI:** 10.1186/s13073-015-0252-1

**Published:** 2015-12-18

**Authors:** Chandan Kumar-Sinha, Shanker Kalyana-Sundaram, Arul M. Chinnaiyan

**Affiliations:** Michigan Center for Translational Pathology, University of Michigan Medical School, Ann Arbor, MI 48109 USA; Department of Pathology, University of Michigan Medical School, Ann Arbor, MI 48109 USA; Department of Computational Medicine and Bioinformatics, University of Michigan Medical School, Ann Arbor, MI 48109 USA; Howard Hughes Medical Institute, University of Michigan Medical School, Ann Arbor, MI 48109 USA; Department of Urology, University of Michigan Medical School, Ann Arbor, MI 48109 USA; Comprehensive Cancer Center, University of Michigan Medical School, Ann Arbor, MI 48109 USA

## Abstract

Enabled by high-throughput sequencing approaches, epithelial cancers across a range of tissue types are seen to harbor gene fusions as integral to their landscape of somatic aberrations. Although many gene fusions are found at high frequency in several rare solid cancers, apart from fusions involving the ETS family of transcription factors which have been seen in approximately 50 % of prostate cancers, several other common solid cancers have been shown to harbor recurrent gene fusions at low frequencies. On the other hand, many gene fusions involving oncogenes, such as those encoding ALK, RAF or FGFR kinase families, have been detected across multiple different epithelial carcinomas. Tumor-specific gene fusions can serve as diagnostic biomarkers or help define molecular subtypes of tumors; for example, gene fusions involving oncogenes such as *ERG*, *ETV1*, *TFE3, NUT*, *POU5F1*, *NFIB*, *PLAG1*, and *PAX8* are diagnostically useful. Tumors with fusions involving therapeutically targetable genes such as *ALK*, *RET*, *BRAF*, *RAF1*, *FGFR1–4*, and *NOTCH1–3* have immediate implications for precision medicine across tissue types. Thus, ongoing cancer genomic and transcriptomic analyses for clinical sequencing need to delineate the landscape of gene fusions. Prioritization of potential oncogenic “drivers” from “passenger” fusions, and functional characterization of potentially actionable gene fusions across diverse tissue types, will help translate these findings into clinical applications. Here, we review recent advances in gene fusion discovery and the prospects for medicine.

**Electronic supplementary material:**

The online version of this article (doi:10.1186/s13073-015-0252-1) contains supplementary material, which is available to authorized users.

## Introduction

Recurrent chromosomal rearrangements in cancers have been described for over half a century [1, 2]. The characterization of the oncogenic fusion *BCR-ABL1* at t(9,22) translocation loci in chronic myeloid leukemia, which culminated in the development of a molecularly targeted therapy, provides a compelling “bench to bedside” paradigm for cancers [3, 4]. Numerous gene fusions have since been defined at cytogenetically distinct loci of recurrent chromosomal aberrations in hematological malignancies and sarcomas, as well as in solid cancers, albeit much less frequently, arguably owing to technical limitations in resolving karyotypically complex, heterogeneous sub-clones in solid tumor tissues [5, 6]. The serendipitous discovery of ETS family gene fusions in common prostate carcinoma [7, 8], and of ALK and ROS kinase fusions in lung cancer [9, 10] through transcriptomic and proteomic approaches, bypassing chromosomal analyses, provided a strong fillip to the search for gene fusions in common solid cancers and pointed to alternative approaches to gene fusion discovery. Developments in high-throughput sequencing techniques over the past decade [11] have made possible a direct, systematic discovery of gene fusions in solid cancers [12–14], rapidly revealing a diverse genomic landscape. Gene fusions have now been identified in several common carcinomas, including those of the prostate, lung, breast, head and neck, brain, skin, gastrointestinal tract, and kidney, which alongside the widely documented gene fusions in thyroid and salivary gland tumors support the notion that gene fusions are integral to the genomic landscape of most cancers.

Here, we review the emerging landscape of gene fusions across solid cancers, focusing on the recent spurt of discoveries made through sequencing. We review common features of “driver” fusions (those that contribute to tumor progression), the major functional classes of fusions that have been described, and their clinical, diagnostic and/or therapeutic implications.

## Detection of gene fusions in carcinoma

The first gene fusions to be defined in solid cancers, *RET*/*PTC* [15] and *NTRK1* [16] rearrangements in papillary thyroid carcinoma were identified through a “transformation assay” using cancer genomic DNA transfected into murine NIH3T3 cells, followed by retrieval and analysis of human genomic DNA from transformed cells [17]. More typically, karyotyping and cytogenetic analysis of recurrent translocations helped define early gene fusions in solid cancers, such as *CTNNB1-PLAG1* [18] and *HMGA2* fusions [19] in salivary gland pleomorphic adenomas, *PRCC-TFE3* in renal cell carcinomas [20], and *ETV6-NTRK3* fusion in secretory breast carcinoma [21]. Incorporating more molecular approaches, a recurrent 2q13 breakpoint locus, t(2;3)(q13;p25), in follicular thyroid carcinoma was fine mapped using yeast artificial chromosomes, and cloned through 3′ rapid amplification of cDNA ends (RACE) of the candidate *PAX8* cDNA, leading to characterization of the *PAX8-PPAR*γ gene fusion [22]. Anticipating high-throughput genomics approaches, an expressed sequence tag (EST) mapping to the recurrent chromosomal breakpoint at t(15;19)(q13;13.1) in midline carcinoma was identified from an EST database and cloned through RACE to identify the pathognomonic gene fusion *BRD4-NUT* [23]. The gene fusions defined in solid cancers thus far were localized at cytogenetically distinct, recurrent chromosomal aberrations, and were largely confined to relatively rare subtypes of solid cancers [5].

**Fig. 1:**
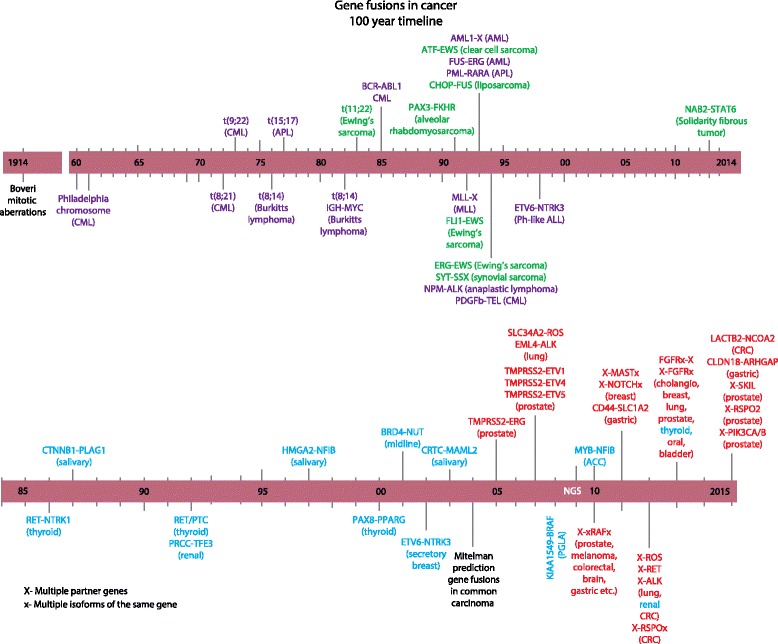
Timeline of gene fusion discoveries. A timeline representation of salient gene fusion discoveries starting with 1914, the year that marked the publication of Boveri’s monograph “*Zur Frage der Entstehung maligner Tumoren*”, in which he proposed that aberrant “combinations of chromosomes” underlie malignant transformation [25]. The *top bar* shows recurrent chromosomal rearrangements or gene fusions in hematological (*purple*) and soft tissue (*green*) malignancies, and the *bottom bar* shows gene fusions in relatively rare (*blue*) and those in common (*red*) epithelial cancers. *ACC* adenoid cystic carcinoma, *AML* acute myeloid leukemia, *ALL* acute lymphoblastic leukemia, *APL* acute promyelocytic leukemia, *cholangio* cholangiocarcinoma, *CML* chronic myeloid leukemia, *CRC* colorectal carcinoma, *MLL* mixed lineage leukemia, *PLGA* pediatric low grade astrocytoma, *Ph* Philadelphia chromosome

However, between 2005 and 2007, independent of a priori evidence of genomic rearrangements, recurrent gene fusions involving ETS family genes were discovered in prostate cancer, based on analysis of genes displaying outlier expression [7, 8, 24]. Around the same time, a transformation assay with a cDNA expression library (*not* genomic libraries [17]) from a lung adenocarcinoma sample led to the discovery of *EML4-ALK* fusions [10], and a high-throughput phosphotyrosine signaling screen of lung cancer cell lines and tumors identified *SLC34A2-ROS1* fusions in non-small-cell lung carcinoma (NSCLC) [9]. Thus, analyses of cancer RNA and proteins provided a critical breakthrough in the identification of oncogenic gene fusions in common carcinoma. In Fig. 1, we summarize the timeline of gene fusion discoveries, 100 years since Boveri’s prescient hypothesis that malignant tumor growth is a consequence of chromosomal abnormalities, including “combinations of chromosomes” [25].

### Next-generation sequencing

High-throughput sequencing of tumor samples provides a direct readout of chimeric sequences corresponding to putative gene fusions, and the available depth of coverage helps uncover even relatively minor, sub-clonal events. In a proof of principle study, high-throughput genomic sequencing was used to identify several gene fusions in a panel of breast cancer cell lines and tissues [14]. However, considering that only a small subset of genomic breakpoints correspond to gene fusions encoding fusion transcripts or proteins, alternative approaches were explored. In a directed approach, focusing on chimeric transcripts as the readout of “expressed” gene fusions, Maher and colleagues used coupled short- and long-read transcriptome sequencing [12] and paired-end transcriptome sequencing [13] to detect chimeric RNAs that could be analyzed to characterize gene fusions. RNA sequencing has since been widely used in the discovery of numerous gene fusions in diverse epithelial cancers. Additionally, paired-end tag [26] and chromatin interaction analysis by paired-end-tag sequencing have been employed for gene fusion discovery [27], as well as phosphoproteome analysis, as in the discovery of a *SND1-BRAF* fusion in a gastric carcinoma sample [28]. The DNA- or protein-based methods, however, are not as commonly used as RNA sequencing, likely owing to several additional, specialized steps that are involved.

Interestingly, RNA sequencing has also identified a class of chimeric RNAs that do not involve chromosomal aberrations. For example, “read-through” chimeric *SLC45A3-ELK4* transcripts, such as those detected in prostate cancer, result from runaway transcription of the androgen-inducible, prostate-specific gene *SLC45A3* into *ELK4*, the adjacent ETS family gene in the same orientation [12, 29–31]. Similarly, the *VTI1A*-*TCF7L2* fusion, originally identified through genomic sequencing of colorectal carcinoma (CRC) samples [32], was found in a follow-up study using RNA analyses to be quite prevalent in other cancers, as well as in benign samples [33]. Chimeric transcripts not associated with genomic translocation have also been observed between non-contiguous genes. Guerra and colleagues identified *CCND1*-*TACSTD2* (*TROP2*) chimeric mRNA that involves genes located on different chromosomes in subsets of ovarian, breast, gastrointestinal, and endometrial cancers [34]. The functional significance of these RNA chimeras is not clear at present, as their expression is typically seen to be relatively non-specific.

### Driver and passenger gene fusions

High-throughput sequencing of cancer samples frequently identifies multiple gene fusions in individual samples, often presenting a challenge for identifying potentially oncogenic driver fusions among irrelevant passenger aberrations. Some useful generalizations have emerged from multiple analyses: first, driver fusions are typically marked by a continuous open reading frame (ORF) that retains functional domains, such as the kinase domain in gene fusions involving oncogenic kinases, or DNA-binding domains in the case of transcription factors; second, some fusions display loss of auto-inhibitory domains (for example, loss of the N-terminal inhibitory domain in the product of *BRAF* fusions, or loss of 3′ UTR sequences in *FGFR* or *HMGA2* fusions that serve as binding sites for inhibitory microRNAs). Yet other types of fusions juxtapose the promoter of certain tissue-specific, inducible or highly expressed genes; for example, the prostate-specific, androgen-inducible genes *TMPRSS2* or *SLC45A3* fused in frame with the proto-oncogenes *ERG* or *BRAF*, respectively, generate the *TMPRSS2-ERG* and *SLC45A3-BRAF* gene fusions in prostate cancer.

In the case of novel gene fusions involving less characterized genes, distinguishing candidate driver fusions from random events is complicated by the many false positive candidates resulting from alignment artifacts, such as multi-mapping of reads owing to homologous (pseudogenes) and/or repetitive sequences, and sequencing artifacts due to errors in library generation (particularly ligation and PCR artifacts) and sequencing. Incorporating these considerations, and additional bioinformatics filters, various bioinformatics pipelines have been developed to help prioritize fusion candidates from next-generation sequencing (NGS) data, including Chimerascan [35], FusionSeq [36], DeFuse [37], TopHat-Fusion [38], PRADA [39], and JAFFA [40]. While useful to help reduce the number of false candidates, the output from bioinformatics pipelines needs to be further validated, preferably followed by functional assays, before designating candidate gene fusions as novel driver aberrations. Recurrence of fusions, fusion partners or partner gene families in gene fusion databases also helps to prioritize candidate fusions. Once validated, screening for novel gene fusions in larger cohorts of samples employs quantitative RT-PCR or more recent techniques such as nano-string-based detection [41–43].

## Overview of the landscape of gene fusions in epithelial cancers

From the first reported chromosomal rearrangements in the 1960s up to the year 2000 (roughly marking the advent of high-throughput molecular techniques), the Mitelman Database of Chromosome Aberrations and Gene Fusions in Cancer catalogued more than 600 “recurrent balanced neoplasia-associated aberrations”, in which solid cancers accounted for less than 20 % [44]; in its latest update (7 May 2015), this database lists 10,004 “gene fusions” [45], with solid cancers accounting for a much greater proportion, and with a large number of these fusions identified by recent high-throughput gene expression or sequencing analyses. Over the last decade, numerous gene fusions have been characterized in diverse solid cancers, including ETS family gene fusions in prostate cancer [7, 8, 12, 30, 46–56]; ALK, ROS1 and RET kinase fusions in lung cancer [9, 10, 57–69]; RAF kinase fusions in brain tumors [70–80], melanoma [81, 82], gastric cancer [28, 82], and prostate cancer [82, 83]; R-spondin fusions in colorectal and prostate cancer [83, 84]; *CD44-SLC1A2* gene fusions in gastric cancer [85]; MAST- and NOTCH-family gene fusions in breast cancer [86]; *MITF* gene fusions in renal cancer [87]; and a number of FGFR family fusions in diverse cancer types [88] (Table 1). More than 8000 gene fusions across 16 different tumor types are tabulated in The Cancer Genome Atlas (TCGA) Fusion gene Data Portal (http://www.tumorfusions.org) [89]. The key points regarding gene fusions in epithelial cancers are summarized in Box 1.

**Table 1:** Recurrent gene fusions in epithelial cancers of different body tissues and their role as clinical biomarkers

Tissue or body region	Tumor type	Aberration	Genetic alteration	Diagnostic/prognostic/therapeutic significance	Reference
Thyroid gland	Papillary thyroid cancer (PTC) (>80 % of thyroid cancers)	*RET* gene fusions	Multiple different 5′ partners (most common being *CCDC6* (*PTC1*) and *NCOA4* (*PTC3*)) fuse to 3′ partner *RET*	10–30 % of PTC cases. RET is an oncogenic receptor tyrosine kinase sensitive to FDA-approved drugs, including vandetanib and cabozantinib	[15]
		*NTRK1* gene fusions	5′ activating gene partners including *TPM3*, *TPR* and *TGF* fuse with 3′ partner *NTRK1*	5 % of PTC cases. NTRK1 is an oncogenic receptor tyrosine kinase, potentially targetable by kinase inhibitors	[15, 16]
		*ETV6-NTRK3*	Chromosomal translocation t(12;15) (p13;q25) generates the fusion, with the dimerization domain of ETS family transcription factor (TF) ETV6 fused to the tyrosine kinase domain of NTRK3. Involves exon 14 of *NTRK3*, unlike other *ETV6*-*NTRK3* fusions, which involve exon 13	Radiation-associated PTC (14.5 % post-Chernobyl); 2 % of sporadic PTC cases. Second only to *RET* fusions in prevalence	[121]
	Radiation-induced PTC	*AKAP9-BRAF*	In-frame fusion between exons 1–8 of the *AKAP9* gene and exons 9–18 of BRAF protein kinase gene, lacking the auto-inhibitory N-terminal domain	Fusion-positive tumors lack *BRAF-*activating point mutations. Fusion causes constitutive activation of BRAF and downstream MAPK pathways. Thus, a potential target for MEK inhibitors	[70]
	Follicular thyroid carcinoma (FTC) (10–20 % of thyroid cancers)	*PAX8-PPAR*γ	Chromosomal translocation t(2;3)(q13;p25) results in chimeric protein involving the DNA-binding domain of the thyroid-specific TF PAX8 fused to PPARγ	Fusion-positive FTCs appear to have a significantly better prognosis compared with those lacking this fusion. FTC cells expressing PAX8–PPARγ fusion protein show reduced tumor progression in a mouse xenograft model	[22, 122]
Head and neck	Pleomorphic adenoma	*PLAG1* gene fusions	Multiple 5′ partners (*CHCHD7, CTNNB1, FGFR1, LIFR, TCEA1*) fuse to 3′ *PLAG1*	*PLAG1* encodes a zinc finger TF that regulates IGF2 mitogenic signaling pathway	[18, 91]
		*HMGA2* gene fusions	*HMGA2* is fused with different 3′ partners (including *FHIT*, *NFIB*, and *WIF1*)	The fusion retains all the functional domains of HMGA2, and removes the 3′ UTR sequence that contains several inhibitory *let7* microRNA binding sites. Absence of the Let-7-regulated 3′ UTR in the fusion transcript results in overexpression of HMGA2 that is sufficient for neoplastic transformation	[19]
		*FGFR-PLAG1*	*FGFR* is the 5′ partner, which, without its kinase domain, provides the promoter to drive the expression of the 3′ partner, *PLAG1*	This *FGFR* fusion product does not include the FGFR kinase domain, and therefore is not a target for FGFR inhibitors	[91]
	Adenoid cystic carcinomas (salivary glands, lacrimal glands, ceruminal glands; also breast)	*MYB-NFIB*	Inter-chromosomal gene fusion generating a chimeric transcript comprising almost the entire reading frame of the *MYB* oncogene fused to the last two exons of *NFIB*	*MYB* likely provides the oncogenic activity, while *NFIB* primarily replaces a potentially inhibitory 3′ UTR of *MYB*	[119, 120]
	Acinic cell carcinoma, cystadenocarcinoma, mammary analogue secretory carcinoma of salivary glands (MASC)	*ETV6-NTRK3* (*TEL-TRKC*)	Chromosomal translocation t(12;15) (p13;q25) generates the *ETV6-NTRK3* fusion, with the dimerization domain of the ETS family TF ETV6 fused to the tyrosine kinase domain of NTRK3	This fusion is now considered pathognomonic of MASC	[103]
	Mucoepidermoid carcinoma (MEC) in the oral cavity (also lung, cervix and thyroid glands, and clear cell hidradenoma of skin)	*CRTC1-MAML1* or *CRTC3-MAML2*	Generated by chromosomal translocation t(11;19)(q14–21;p12–13). The product of the 3′ partner *MAML2* acts as a co-activator of NOTCH independent of NOTCH ligand to impart the oncogenic phenotype.	The *CRTC*-*MAML2* fusion is restricted to MEC and has been associated with favorable prognosis.	[107, 108, 110–114, 116, 117]
Midline anatomical structures	Nut midline carcinoma (NMC)	*BRD*-*NUT*	75 % of NMCs express BRD4-NUT fusion proteins, the rest harbor *BRD3* or other 5′ partner genes fused to *NUT*. BRD-NUT fusion proteins contain the N-terminal BET bromodomain, extraterminal domain, and nuclear localization signal fused to the entire coding region of NUT protein that contains a histone acetyltransferase binding domain	NMC is a rare but aggressive squamous cell carcinoma originating from midline anatomical structures such as the head, neck or mediastinum (including the bladder, thymus, lung, and skeleton) that is defined by the presence of *BRD*-*NUT* fusions. BRD proteins have recently emerged as promising therapeutic targets	[104, 105]
Kidney	Renal cell carcinoma (RCC)	*TFE3* gene fusions	Translocations at the Xp11.2 breakpoint result in gene fusions involving the *TFE3* gene with various 5′ partners (*ASPSCR1, PRCC, NONO, CLTC,* and *SFPQ*)	15 % of patients with RCC aged <45 years have this aberration. Fusion-positive RCCs in older patients are more aggressive	[20, 87]
		*ALK* fusions	In *VCL-ALK* fusions*,* the 3′ portion of the *ALK* transcript encoding the kinase domain is fused in frame to the 5′ portion of *VCL*	Found in pediatric RMC that affects young black individuals with the sickle cell trait. In two independent reports, RMC tumors from three cases of African–American children with sickle cell anemia were found to harbor the *VCL-ALK* fusion	[125, 126]
	Non-clear cell renal cell carcinoma (nccRCC)	*CLTC*-*TFEB*	This encodes an in-frame fusion protein containing the conserved bHLH domain of TFEB (similar to other fusions involving *TFEB*), and is associated with the “MITF high” phenotype	Associated with high expression of the anti-apoptotic protein BIRC7, thus potentially sensitive to apoptosis-sensitizing BIRC7 inhibitors that are under development	[88]
		*ACTG1*-*MITF*	In this fusion protein the first 118 amino acids of MITF are replaced by the N-terminal 121 amino acids of ACTG1	Although found in only one sample, ectopic expression of the *ACTG1*-*MITF* fusion led to cellular transformation, suggesting a potential driver function	[87]
Prostate	Prostate cancer	*TMPRSS2*-*ERG*	The 5′ partner *TMPRSS2* contributes prostate-specific, androgen-inducible upstream regulatory elements fused to the 3′ partner, encoding oncogenic ETS family TF ERG	Probably the most prevalent gene fusion in epithelial carcinoma, with 40–50 % of localized prostate cancers found to harbor this fusion across multiple independent cohorts around the world. Associated with prostate carcinogenesis and distinct clinical correlates compared with fusion-negative prostate cancers	[7, 8, 46–52]
		Fusions involving other ETS family genes, including *ETV1*, *ETV4*, *ETV5*, *ELK4,* and *FLI1*	5′ partners include androgen-inducible genes such as *TMPRSS2*, *SLC45A3*, and *FLJ35294*, and androgen-repressed *C15ORF21*, or housekeeping genes such as *HNRPA2B1* and *DDX5*, fused to multiple 3′ oncogenic ETS family TF genes	Together these represent 10–20 % of localized prostate cancers	[7, 8, 24, 53–56]
		*RAF* gene fusions (*SLC45A3*-*BRAF* and *ESRP1*-*RAF1*)	*SLC45A3* is a prostate-specific, androgen-inducible gene fused upstream to gene encoding N-terminal-truncated BRAF, resulting in constitutive activation of this potent oncogene	Although rare, *BRAF*/*RAF1* fusions represent therapeutic targets	[82, 83]
		*TMPRSS2-SKIL, SLC45A3-SKIL, MIPEP-SKIL, PIPOL1-SKIL, ACPP-SKIL, HMGN2P46-SKIL*	5′ partners *TMPRSS2*, *SLC45A3*, and *ACPP* contribute prostate-specific, androgen-inducible upstream regulatory elements fused to 3′ partner *SKIL*, a negative regulator of SMAD	SKIL fusions are observed in 1–2 % of prostate cancers and potentially upregulate the TGF-β pathway	[101]
		*TBXLR1-PIK3CA, ACPP-PIK3CB*	Index cases with PIK3CA/B fusions show outlier expression of PIK3CA/B. ACPP imparts androgen-responsive expression to PIK3CB	PIK3CA fusions may be responsive to PIK3CA inhibitors	[83]
		*GRHL2-RSPO2*	Index cases with RSPO2 fusions/rearrangements show outlier expression of RSPO2	RSPO2 is an agonist of the Wnt pathway and therefore may be responsive to porcupine inhibitors	[83, 142]
Lung	Lung cancer	*ALK* gene fusions (most commonly *EML4-ALK*, but also *TFG-ALK*)	EML4-ALK fusion encodes the N-terminal portion of EML4 fused to the intracellular portion of ALK, always retaining the tyrosine kinase domain	EML4-ALK fusion is reported in 3–7 % of patients with NSCLC in different cohorts. ALK-fusion-positive lung cancers are sensitive to the FDA-approved kinase inhibitor crizotinib	[10, 59–63, 65, 66]
		*ROS1* gene fusions	Multiple 5′ partners such as *TPM3*, *SDC4*, *SLC34A2*, *CD74*, *EZR*, *LRIG3*, and *GOPC* fused to *ROS1*. All of the fusion proteins retain the kinase domain of ROS1	2 % of lung cancer samples in one study	[9, 43, 58, 65]
		*RET* gene fusions	Multiple isoforms of *KIF5B-RET* and *CCDC6-RET*. All of these products retain the kinase domain of RET	Lung cancer cases with RET fusions may be candidates for FDA-approved RET inhibitor therapies such as vandetanib and cabozantinib	[64–69]
Mammary gland	Breast cancer	*ETV6-NTRK3* (*TEL-TRKC*)	Chromosomal translocation t(12;15) (p13;q25) generates *ETV6-NTRK3* fusion, with the dimerization domain of the ETS family TF ETV6 fused to the tyrosine kinase domain of NTRK3	Almost 100 % of secretory breast carcinomas. ETV6-NTRK3 chimeric protein activates the IRS1 adapter protein, RAS-MAP kinase and PI3K-AKT pathways, and suppresses TGF-β signaling. ETV6-NTRK3-expressing cells and tumors are sensitive to the IGIFR/INSR kinase inhibitors BMS-536924 and BMS-754807 (currently in clinical trials)	[21, 142]
		*MAST1* and *MAST2* gene fusions	5′ partners including *ZNF700, NFIX,* and *TADA2A* fused to *MAST1. ARID1A* and *GPBP1L1* fused to *MAST2* serine/threonine kinase. All *MAST* fusions encode contiguous open reading frames, some retaining the canonical serine/threonine kinase domain, all retaining the PDZ domain and a 3′ kinase-like domain	3 % of breast cancer samples in one study	[86]
		*NOTCH* gene fusions	*SEC16A-NOTCH1, SEC22B-NOTCH2, NOTCH1* exon 2–exon 28 (intramolecular rearrangement)	NOTCH fusions retain the NOTCH intracellular domain, which mediates downstream NOTCH signaling. The SEC16A-NOTCH1 fusion retains the γ-secretase cleavage site and shows sensitivity to γ-secretase inhibitors compared with SEC22B-NOTCH2, which loses this site	[86]
		*EML4-ALK*	*EML4* exon 13 fused to *ALK* exon 20, similar to NSCLC fusions	One exon array profiling study reported *EML4-ALK* fusions in 2.4 % of breast carcinomas (5 of 209). One *EML4-ALK* fusion was detected in inflammatory breast cancer	[82, 124]
Stomach	Gastric cancer	RAF gene fusions	*AGTRAP-BRAF*: N-terminal protein AGTRAP fused to the C-terminal kinase domain of BRAF. *SND1-BRAF*: 5′ *SND1* gene fused to *BRAF*, found in GTL16 gastric cancer cell line	Both these fusions retain the kinase domain of BRAF, indicating potential responsiveness to RAF/MEK inhibitors	[28, 82]
		*CLDN18-ARHGAP26*	*CLDN18* on 3q22.3 fused to *ARHGAP26* on 5q31.3. The fusion protein loses the PH domain of ARHGAP26, but retains the Rho-GAP and SH3 domains	3 % of Southeast Asian gastric cancers	[27]
		*CD44-SLC1A2*	Fusion involving adjacent genes (lying in opposite orientations on chromosome 13p)	1–2 % of gastric cancers	[85]
Gut	Colorectal cancer (CRC)	*EIF3E-RSPO2, PTPRK-RSPO3*	Both these fusion proteins retain the functional domain of the R-spondins, which are known to be agonists of the canonical Wnt/β-catenin signaling pathway	Recurrent fusions involving R-spondin family genes, *EIF3E-RSPO2* (two cases) and *PTPRK-RSPO3* (five cases) were detected by RNA sequencing of 68 “microsatellite stable” subtype CRC samples	[85]
		*LACTB2-NCOA2*	The fusion disrupts expression of NCOA2, which is an inhibitor of the Wnt/β-catenin pathway. This loss-of-function fusion thus represents a novel oncogenic mechanism in a subset of CRC	Found in 6 of 99 (6.1 %) CRC cases	[103]
		*VTI1A-TCF7L2, RP11-57H14.3- TCF7L2*	Gene fusion involving activator of Wnt/β-catenin signaling pathway. VTI1A-TCF7L2 fusion lacks the TCF4 β-catenin-binding domain	*VTI1A-TCF7L2* was found in 3 of 97 CRCs. A screen for *TCF7L2* fusion transcripts revealed its presence in more than 80 % of CRCs, 29 % of normal colonic mucosa, and 25–75 % of normal tissues from other organs. Thus, *TCF7L2* fusion transcripts are neither specific to cancer nor to the colon or rectum. *TCF7L2* fusion transcripts represent “read through” events	[32, 33]
Skin	Melanoma	*BRAF* and *RAF1* gene fusions	Diverse N-terminal proteins fused to the BRAF/RAF kinase domain	Seen in 3 % of melanomas; fusions retain the kinase domain of BRAF, indicating potential responsiveness to RAF/MEK inhibitors	[82]
		Other, non- recurrent aberrations	*RB1-ITM2B, PARP1-MIXL1, RECK-ALX3, TMEM8B-TLN1, CCT3-C1orf61, GNA12- SHANK2, ANKHD1-C5orf32*	11 novel gene fusions were identified in 6 different patient samples, including both inter- and intra-chromosomal events. These fusions encode putative dominant-negative proteins (RB1, PARP1), and a truncated inhibitor of tumor invasion and metastasis (RECK)	[81]
Central nervous system	Gliomas	*PTPRZ1-MET*	The fusion involves translocation of introns 3 or 8 of *PTPRZ* and intron 1 of *MET*	Found only in grade III astrocytomas (1/13; 7.7 %) or secondary GBMs (3/20; 15.0 %)	[71]
	Pilocytic astrocytoma	*BRAF*/*RAF1* gene fusions	*KIAA1549-BRAF, FAM131B-BRAF, SRGAP3-RAF1*	Most frequently observed in pediatric brain tumors. Approximately 80 % of pilocytic astrocytomas and other low-grade gliomas harbor the *KIAA1549-BRAF* gene fusion	[72–80]

*FDA* Food and Drug Administration, *FTC* follicular thyroid carcinoma, *GBM* glioblastoma multiforme, *MASC* mammary analog secretory carcinoma of salivary glands, *MEC* mucoepidermoid carcinoma, *nccRCC* non-clear-cell renal cell carcinoma, *NMC* NUT midline carcinoma, *NSCLC* non-small-cell lung carcinoma, *PTC* papillary thyroid cancer, *RCC* renal cell carcinoma, *RMC* renal medullary carcinoma, *TF* transcription factor

**Fig. 2:**
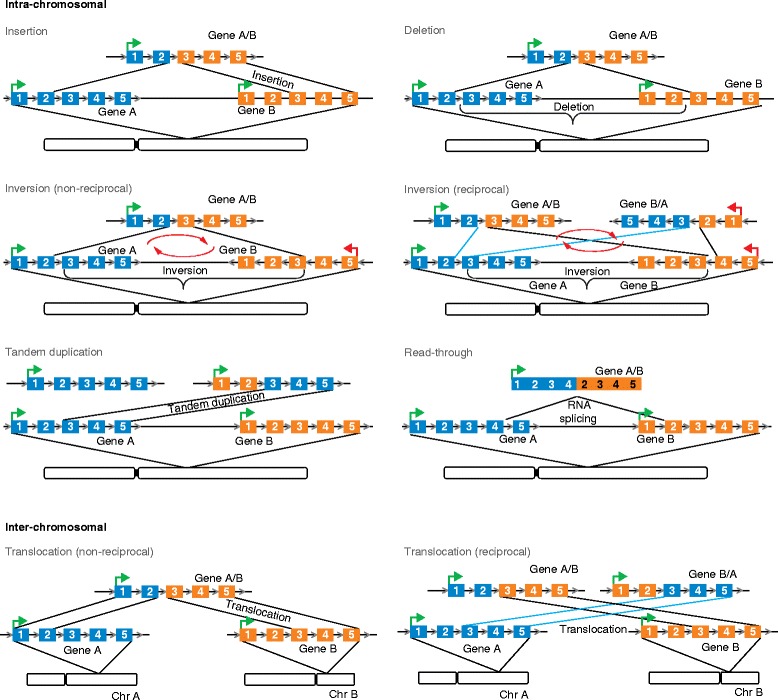
Diversity in the architecture of gene fusions. Schematic representation of different patterns of chromosomal rearrangements inferred from chimeric transcripts. Exons of genes involved in fusions are shown in *blue* and *orange*, and their transcriptional orientation is denoted by *arrows*. The likely mechanisms of chimera generation are indicated. *Chr* chromosome

These gene fusions in solid cancers encompass the diversity of fusion architectures, as shown in Fig. 2 and Box 2, and represent a spectrum of functional categories, including those described earlier such as kinases and transcription factors, as well as those involving newer pathways and loss-of-function fusions (discussed later). Notably, even as numerous novel gene fusions are being discovered fairly rapidly, most of these are either non-recurrent singletons, or are seen to recur at exceedingly low frequency in tumor subtypes or to recur across tumor types (Table 1). Incidentally, gene fusions displaying molecular recurrence involving both 5′ and 3′ partner genes, as in *TMPRSS2-ERG*, *EML4-ALK*, and *BRD4-NUT*, are relatively few. A large number of fusions display recurrence of a fusion gene in combination with multiple different partners; for example, *BRAF*/*RAF1* [76, 79, 82, 83] and *FGFR1*/*2*/*3* [88–94] are fused to several different 5′ partners across different tissue types (Additional file 1). This heterogeneity is likely reflective of the diverse tissue–physiological milieu in which these oncogenes impart selective advantage to the cancer cells. Conversely, some lineage-specific genes are seen to serve as 5′ partners across multiple different 3′ genes; for example, *TMPRSS2* and *SLC45A3* in prostate cancer have been observed as 5′ partners of *ERG*, *ETV1*, *ETV4*, *ETV5*, *BRAF*, and *ELK4* (Table 1 and Additional file 1). Another type of observed “recurrence” involves isoforms of a gene family — for example, *ETV1*/*2*/*3*/*4*/*5*, *FGFR1*/*2*/3, *BRAF*/*RAF1*, *BRD3*/*4*, *CRTC1*/*CRTC3*, and *NTRK1*/*3* — as fusion partners. Considering that individual fusions may be observed relatively rarely (even uniquely), the potential functional consequences of gene fusions assumes priority over considerations of recurrence.

## Functional consequences of gene fusions

Functionally distinct molecular classes of gene fusions that are shared across tumor types can be identified in solid cancers.

### Kinases

Given their therapeutic importance, identification of gene fusions involving kinases can often signify a clinically actionable observation. Kinase fusion genes detected across multiple cancer types include *RET*, *NTRK1*, *NTRK3*, *ALK*, *ROS1*, *FGFR1*/*2*/*3*, and serine threonine kinases including the RAF family genes *BRAF*, *RAF1*, *CRAF*, and *MAST1*/*2* (Table 1 and Additional file 1). In most gene fusions involving kinases, the kinase domain is retained [95], and this provides a strong filtering criterion in high-throughput sequencing data analysis. Analysis of mRNA sequencing data from the TCGA compendium, comprising 4366 primary tumor samples from 13 tissue types, revealed kinase fusions involving *ALK*, *ROS*, *RET*, *NTRK*, and *FGFR* gene families, which were detected in several types of cancer: bladder carcinoma (3.3 %), glioblastoma (4.4 %), head and neck cancer (1.0 %), low-grade glioma (1.5 %), lung adenocarcinoma (1.6 %), lung squamous cell carcinoma (2.3 %), and thyroid carcinoma (8.7 %) [89].

### Transcription factors

Gene fusions involving dysregulated expression of transcription factors include ETS family gene fusions, seen in approximately 50 % of all prostate cancers and probably one of the most prevalent transcription factor gene fusions in common epithelial cancers. Among these, *ERG* represents the most common fusion partner and *ETV1* the most promiscuous, with a dozen or more different fusion partners described to date (Additional file 1) [24, 96].

Other gene fusions involving transcription factors include *NUT* (or *NUTM1*), *POU5F1*, *MAML2*, *NFIB*, *PLAG1*, *TFE3*, *NOTCH*, and *PAX8* fusions, imparting spatially and/or stochastically dysregulated expression in multiple different cancer types. *NOTCH1* and *NOTCH2* fusions result in dysregulated transcriptional outcomes, because after ligand activation, the NOTCH intracellular domain (NICD) forms a transcriptional activator complex, activating genes involved in differentiation, proliferation and apoptosis, and those associated with carcinogenesis. MAML2 acts as a transcriptional co-activator for NOTCH proteins by amplifying NOTCH-induced transcription of *HES1*. TFE3, which belongs to the MITF/TFE family of basic helix-loop-helix leucine zipper transcription factors, is involved in TGF-β-induced transcription, and has important roles in cell growth and proliferation. *TFE3* is involved in chromosomal translocations that result in various gene fusions (such as *PRCC*-*TFE3*, *RCC17*-*TFE3*, *PSF*-*TFE3*, *NONO(p54nrb)-TFE3* and *ASPL*-*TFE3*) in papillary renal cell carcinomas. PLAG1 is an oncogenic transcription factor associated with the neoplastic transformation of pleomorphic adenomas of the salivary gland and lipoblastomas through upregulation of *IGF2*, *CRLF1*, *CRABP2*, *CRIP2*, and *PIGF*. NFIB binds viral and cellular promoters activating transcription and replication. POU5F1 and PAX8 are homeobox-containing transcription factors, a family of genes that play a role in cell fate and differentiation programs, and whose role in cancer is well recognized, particularly *PAX8* in thyroid cancer [22].

### Other functional classes

#### Metabolic enzymes

*CD44-SLC1A2*/*EAAT2* gene fusions are detected in 1–2 % of gastric cancers involving the glutamate transporter *SLC1A2* [85], and cause intracellular accumulation of glutamate, a growth-promoting amino acid associated with oncogenic functions [97, 98]. Thus, this gene fusion may be establishing a pro-oncogenic metabolic milieu, akin to the increased levels of sarcosine reported in prostate cancer [99].

#### Wnt/β-catenin signaling pathway

RNA sequencing of 68 “microsatellite stable” subtype colorectal cancer samples revealed two recurrent fusions involving R-spondin family genes, *EIF3E*-*RSPO2* in two cases and *PTPRK*-*RSPO3* in five cases [84]. Both these gene fusions retained the functional domain of the R-spondins that are known to be agonists of the canonical Wnt/β-catenin signaling pathway. Additionally, the *LACTB2*-*NCOA2* chimeric transcript detected in 6 of 99 (6.1 %) colorectal cancer cases led to disruption of *NCOA2* expression, thus activating the Wnt/β-catenin pathway [100]. Recently, R-spondin fusions such as *GRHL2-RSPO2* were described in prostate cancer as well [83].

#### TGF-β pathway

Recently, fusions involving *SKIL* (which encodes a SMAD inhibitor) 3′ to androgen-regulated promoters such as *TMPRSS2*, *SLC45A3*, and *ACPP*, were found in 6 of 540 (1.1 %) prostate cancers and one cell line xenograft, LuCaP-77 [101]. *SKIL* overexpression in these tumors was associated with upregulation of the TGF-β pathway, likely providing the oncogenic mechanism in these tumors.

#### Chromatin modifier genes

In an analysis of fusion transcripts observed in TCGA data across multiple tumor types, fusions involving chromatin modifier genes, including histone methyltransferase and histone demethylase genes, were identified in 111 samples (2.5 %) [89]. Chromatin modifier genes are potential therapeutic targets and these gene fusions thus represent a novel class of potentially actionable aberrations.

#### Further functional classes

Additional classes of genes represented among recurrent fusions in solid cancers include those encoding growth factor receptors (*GABBR2*, *TACSTD2*, *ITPR2*), adaptors and co-factors (*WIF1*, *GAB2*), Ras-Gap proteins (*DOCK5*, *ARHGAP15*), and cytoskeletal proteins (*SNF8*, *SEC22B*, *HIP1R*, *STXBP4*, *MYO19*, *TPR*). Although some of these fusions are scored as recurrent, they may represent passenger mutations associated with loci of recurrent chromosomal aberrations, while others may define tissue-specific or cooperative roles.

### Loss-of-function gene fusions

While most reported gene fusions pertain to gain-of-function aberrations imparting neoplastic phenotypes, with high-throughput sequencing, fusions resulting in loss of function of tumor suppressors such as *TP53* and *PTEN* have been identified as well [102]. The *LACTB2*-*NCOA2* fusion in colorectal cancer leads to disruption of *NCOA2*, which encodes an inhibitor of the Wnt/β-catenin pathway [100], thus acting to promote carcinogenesis.

## Gene fusion signatures in personalized medicine of epithelial cancers

Some gene fusions are associated with distinct subtypes of carcinoma, while others have been detected across different tissues or lineages, defining molecular subsets of cancers transcending morphological distinctions.

### Recurrent gene fusions as biomarkers of subtypes of solid cancers

Some of the salient gene fusions that define molecular subtypes of epithelial cancers within specific organs or tissue types are summarized in Table 1. The *ETV6*-*NTRK3* fusion is a diagnostic biomarker of secretory breast carcinoma, as well as the acinic cell carcinoma or cystadenocarcinoma recently designated as “mammary analog secretory carcinoma of salivary glands” (MASC) [21, 103]. *BRD-NUT* fusions define NUT midline carcinoma [104, 105]. *CRTC*-*MAML2* fusions are the defining molecular aberration of mucoepidermoid carcinoma (MEC) [106, 107]; translocation-negative MECs are proposed to be designated as a distinct subgroup of adenosquamous carcinoma [108]. *CRTC-MAML* fusions are also found in MEC of the lung [109–112], cervix [113], thyroid glands and oral cavity [114], as well as in clear cell hidradenoma of the skin [115, 116]. In all cases, *MAML2* fusions characterize benign or low-grade tumors, and for reasons not described so far have been associated with a favorable prognosis [117]. Interestingly, pulmonary MECs have shown clinical response to gefitinib in the absence of sensitizing *EGFR* mutations, suggesting a potential connection with *CRTC*-*MAML2* and the possibility of therapeutic application in other MECs harboring this fusion [110, 118]. The diagnostic subclass of adenoid cystic carcinomas, including salivary gland and breast cancer, is characterized by *MYB-NFIB* gene fusions [119, 120]. Fusions defining subtypes within a cancer include *RET* and *NTRK* gene fusions in subsets of papillary thyroid carcinoma [121], while *PAX8-PPAR*γ fusions characterize subsets of follicular thyroid carcinoma [22, 122]. ETS family gene fusions, primarily including *ERG* (and less frequently, *ETV1*, *ETV4*, *ETV5* or *FLI1*), are found in approximately 50 % of prostate cancers, the most common fusion being *TMPRSS2-ERG*. The *EWSR1*-*ATF1* fusion found in hyalinizing clear cell carcinoma of the salivary glands, a rare and indolent tumor, can potentially be used as a molecular marker of this subtype that is histologically similar to the more aggressive MEC [123].

Gene fusions or fusion partners found across tissue types are common in solid cancers. The *EML4*-*ALK* fusion, initially identified in lung cancer [9, 10] has since been reported in breast cancer [124], colorectal carcinomas [66, 124], and in pediatric renal medullary carcinoma that affects young African–Americans with the sickle cell trait [125, 126]. Similarly, *RET* fusions, first characterized in thyroid cancer, are widely observed in lung cancers, and the *EWSR1*-*POU5F1* fusion was detected in two rare epithelial tumors, hidradenoma of the skin and MEC of the salivary glands [127].

Gene fusions involving RAF kinase genes (*BRAF*, *RAF1*, *CRAF*) have been identified in low-grade tumors of the central nervous system (pilocytic astrocytomas and other low-grade gliomas), gastric cancer, melanoma and prostate cancer. RAF family fusions involve truncation of the N-terminal auto-inhibitory domain, thus generating constitutively active RAF protein. Curiously, *BRAF* gene fusions in low-grade astrocytomas have been associated with a tendency to growth arrest, conferring a less aggressive clinical phenotype and a better clinical outcome [75, 128]. Additionally, RAF family fusions have been defined across diverse solid cancers, including prostate, gastric, and skin cancers [82, 83]. A screen for *BRAF* gene fusions in 20,573 solid tumors, using the FoundationOne™ targeted gene panel, identified *BRAF* fusions involving 29 unique 5′ fusion partners in 55 (0.3 %) cases across 12 different tumor types, including 3 % (14/531) of melanomas, 2 % (15/701) of gliomas, 1.0 % (3/294) of thyroid cancers, 0.3 % (3/1,062) of pancreatic carcinomas, 0.2 % (8/4,013) of non-small cell lung cancers and 0.2 % (4/2,154) of colorectal cancers, as well as single cases of head and neck cancer, prostate cancer, rectal adenocarcinoma, ovarian, uterine endometrial, and mesothelioma [70].

Fusions involving FGFR tyrosine kinase family genes have also been observed across diverse cancers [88]. The first *FGFR* fusion observed in epithelial cancers, *FGFR1-PLAG1*, was found in a subset of pleomorphic salivary gland adenomas*,* and involves *FGFR1* as the 5′ partner upstream of *PLAG1*, the known driver of salivary gland tumors [91]. Curiously, this fusion excludes the tyrosine kinase domain of FGFR. Fusions that retain the tyrosine kinase domain of FGFR include *FGFR3*-*TACC3* in glioblastoma [92, 129]. Subsequently, diverse *FGFR* fusions, all retaining the tyrosine kinase domain, have been observed in bladder, lung, breast, thyroid, oral, and prostate cancers, involving *FGFR1*, *2*, or *3* either as the 5′ or 3′ partners [88, 94].

### Some gene fusions provide personalized therapeutic targets

In Additional file 2 we summarize recent clinical trials involving gene fusions in epithelial cancers. The RET inhibitor vandetanib shows antiproliferative activity in *RET*-mutant medullary thyroid cancer (MTC) [130], and was recently approved by the US Food and Drug Administration for treatment of metastatic MTC. Sensitivity to vandetanib was also observed in *RET*-fusion-positive papillary thyroid carcinoma [131] and lung cancer cells [68, 132]. Treatment with Pfizer’s kinase inhibitor crizotinib (PF02341066) led to a dramatic clinical response in *EML4*-*ALK*-positive NSCLC patients [133, 134], as well as in one patient with an *SLC34A2*-*ROS1*-fusion-positive tumor [58]. Unfortunately, resistance is inevitably observed, owing to mutations in the kinase domain [134, 135], or *ALK* gene fusion amplification, *KIT* amplification or increased auto-phosphorylation of *EGFR* [136]. This is representative of the challenge of treating solid cancers and argues for the development of combinatorial therapeutic approaches from the start rather than sequentially, as is the practice currently. RAF or MEK inhibitors represent potential precision therapeutic options for several solid cancers with the diverse RAF family gene fusions described earlier. Several FGFR inhibitors currently in clinical trials represent potential therapeutics for cancers harboring FGFR fusions across multiple cancer types, including bladder cancer, prostate cancer, and others [88, 90, 94, 137]. The rare PIK3C family gene fusions in prostate cancer (for example, *TBXLR1-PIK3CA* and *ACPP-PIK3CB*) show overexpression of the *PI3KC* genes and may be sensitive to PIK3CA inhibitors [83].

For treatment of secretory breast carcinoma expressing the *ETV6*-*NTRK3* fusion, therapeutic targeting of the downstream signaling axis of IGF1R, using the IGIFR/INSR kinase inhibitors BMS-536924 and BMS-754807 that are currently in clinical trials, was found to be effective [138]. Breast cancer cells expressing *NOTCH* fusion products that retain the γ-secretase cleavage site were sensitive to γ-secretase inhibitor (GSI) in culture, and treatment with GSI reduced tumor growth in vivo [86]. On the other hand, breast cancer cells harboring *NOTCH* fusions that encode NICD independent of the γ-secretase cleavage site were insensitive to GSI.

In a recent clinical sequencing study of 102 pediatric cancers, among 37 non-sarcoma solid cancers, several functional gene fusions were identified, including *TFE3* fusions in a colorectal cancer (*SFPQ-TFE3*) and renal cell cancer (*ASPSCR1*-*TFE3*) — both cases were treated with pazopanib, the latter displaying stable disease for 10 months [139].

Efforts to target several other gene fusions are underway. The newly developed bromodomain inhibitors that have shown dramatic efficacy in hematological malignancies [140, 141] are now being tested in multiple clinical trials for NUT midline carcinoma characterized by *BRD3*/*4-NUT* gene fusions, which represent a rare but highly aggressive class of tumors with no effective treatment currently available [104]. Also, the R-spondin fusions observed in colorectal and prostate cancer may be sensitive to Wnt pathway antagonist porcupine inhibitors [142].

Gene fusions involving ETS transcription factors have been utilized in diagnostic applications. A non-invasive assay system has been developed based on the detection of *TMPRSS2*-*ERG* fusion transcripts in urine samples from patients, which in combination with the detection of urine *PCA3* improved the performance of the multivariate Prostate Cancer Prevention Trial risk calculator in predicting cancer on biopsy [143]. Detection of *TMPRSS2*-*ERG* in circulating tumor cells in therapy-naive patients and in castration-resistant prostate cancer patients following treatment suggests potential applications in non-invasive monitoring of the therapeutic response [144]. While therapeutic targeting of transcription factor oncogenes is intrinsically challenging, on the basis of the interaction of *ERG* with the DNA repair enzyme PARP1 and DNA protein kinase DNA-PKc, use of PARP inhibitors was shown to inhibit growth of *TMPRSS2-ERG*-positive prostate cancer xenografts [145]. Additionally, PARP inhibition was associated with radiosensitization of *TMPRSS2*-*ERG*-positive prostate cancer cells [146, 147]. These experimental leads point to possible therapeutic avenues targeting a prevalent gene fusion in a common carcinoma.

## Perspectives and discussion

Genomic or transcriptomic sequencing has virtually supplanted molecular and cytogenetic techniques as the primary modality for discovery of gene fusions, and detection of gene fusions is increasingly incorporated into the standard workflow for genomic characterization of tumors in both research and clinical settings. Transcriptome sequencing has been useful in helping to identify expressed gene fusions based on evidence of the fusion of exon boundaries, but putative promoter fusions that do not generate chimeric transcripts are likely to go undetected. Furthermore, typically recurrent gene fusions characterized in cancers represent gain-of-function events arising from the juxtaposition of cell-type- or lineage-specific regulatory elements and proto-oncogenes, or novel combinations of functional domains derived from two proteins that provide combinatorial or additive functionalities to normal genes. However, NGS data also reveal less frequently described loss-of-function chimeras involving tumor suppressor genes such as *TP53*, *PTEN*, and others. A systematic analysis of loss-of-function gene fusions could identify additional cancer samples with loss of tumor suppressors that might be currently going unreported, and could help broaden our understanding of the role of gene fusions in cancer.

The rapid increase in detection of gene fusions across cancers has spawned multiple discovery and prioritization pipelines to help distinguish bona fide functional gene fusions from random chimeras (and experimental artifacts). However, the development of diverse pipelines following different analysis parameters underscores a need for standardization of the vocabulary and information content in recording and reporting gene fusions, along the lines of the Minimum Information About a Microarray Experiment [148, 149]. Furthermore, even as bioinformatics analyses help prioritize fusion candidates, the “recurrence” of fusion genes and/or retention of functional domains provide the most compelling rationale for functional characterization.

The detection of distinct gene fusions across subtypes of common carcinoma also provides a basis for molecular subclassification of these cancers. Recurrent gene fusions that characterize distinct subtypes of cancers include *BRD4-NUT* in NUT midline carcinoma, *ETV6-NTRK3* in secretory breast carcinoma, *CRTC-MAML2* fusions in mucoepidermoid carcinoma, and RAF family fusions in pilocytic astrocytomas. It is expected that as more and more carcinomas are analyzed by sequencing, additional subclasses may be recognized on the basis of whether the detected molecular aberrations are driver fusions. Importantly, the emerging landscape of gene fusions in solid cancers also reveals many gene fusions involving oncogene families or isoforms that are seen across multiple tumor types or subtypes, for example, fusions involving RAF and FGFR family genes. This supports the notion that a molecular classification of tumors in terms of driver fusions (or SNVs) may complement histopathological descriptions.

Many oncogenes involved in gene fusions (for example, *RET*, *BRAF*, *ALK, NOTCH* or *PIK3CA*/*B*) are also known to harbor activating mutations. However, fusions and mutations tend to be mutually exclusive. This indicates that either fusions or activating mutations can independently provide oncogenic function, and that either of these aberrations may render the tumors sensitive to therapeutic targeting. Thus, for example, MEK inhibitors that have been found to be useful for tumors with a *BRAF* activating mutation may also benefit tumors with the *BRAF* fusion.

The development of technologies that enable the systematic detection of molecular aberrations in cancer has profound clinical implications, as high-throughput sequencing of individual tumor samples is expected to become available as a routine diagnostic modality (as for whole-body PET scans or MRI) in the not-too-distant future. Considering the important diagnostic and therapeutic implications, the integration of approaches for the detection of driver gene fusions into cancer genomics pipelines is crucial for precision cancer medicine.

## Box 1. Summary points

1. Gene fusions are an integral component of the landscape of somatic aberrations in all cancers.
2. Recurrent 5′ fusion genes are generally lineage- and/or cell-type specific.
3. Recurrent 3′ fusion genes in epithelial cancers are usually kinases or transcription factors, similar to the situation in hematological and soft tissue cancers.
4. High-throughput sequencing enables systematic discovery of gene fusions with high sensitivity and precision.
5. High-throughput sequencing often identifies multiple gene fusions in individual samples, presenting a challenge to distinguish oncogenic “driver” from unimportant “passenger” aberrations.
6. Chimeric RNAs expressed independent of chromosomal rearrangements are frequently observed in cancer (and benign) tissues.
7. Functionally recurrent gene fusions provide clinically relevant molecular subclassifications of existing morphological categories of tumors.
8. Functionally recurrent gene fusions that are seen across tissue types define functionally distinct molecular subtypes of cancers.
9. Gene fusions represent personalized therapeutic targets and prognostic and diagnostic markers.

## Box 2. Mechanisms of generation of gene fusions

An overview of the genomic architecture of gene fusions reveals that fusions may result from insertion, deletion, inversion, or tandem duplication or amplification, and may involve the same chromosome (intra-chromosomal) or different chromosomes (inter-chromosomal) (Fig. 2). A majority of chromosomal rearrangements have been associated with intra-chromosomal tandem duplications and amplifications in multiple whole-genome sequencing studies [14, 26, 80, 150]. Micro-homologies and repeat elements have been associated with loci of recurrent break points [151]. In an analysis of RAF family gene fusion breakpoints in low-grade astrocytomas, tandem duplications generated by microhomology-mediated break-induced replication were identified as the mechanism of generation of fusions [74].

**Fig. 3:**
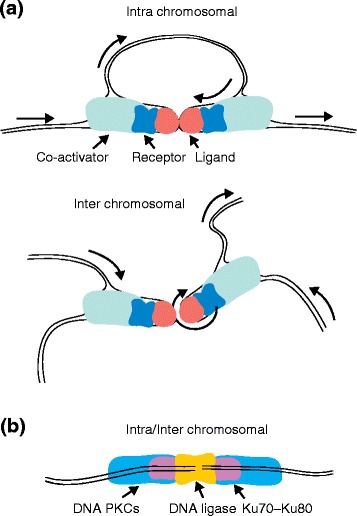
Schematic illustration of the molecular mechanisms underlying the formation of gene fusions. **a** “Induced proximity”, or chromosomal proximity induced by receptor–ligand co-activator-mediated transcription between genes on the same chromosome (intra-chromosomal) or different chromosomes (inter-chromosomal). Physical proximity accompanied by a chromosomal break during transcription or mediated by genotoxic stress can lead to aberrations in DNA repair, which, in turn, may cause the formation of gene fusions. **b** Fusions may result from aberrant DNA double-strand break repair involving alternative-non-homologous end joining machinery. *PKC* protein kinase C

Spatial proximity between distant chromosomal loci has been associated with chromosomal rearrangements, as observed between *RET* and the *H4* genes located 30 megabases (Mb) apart on chromosome 10, involved in *RET* gene fusions in papillary thyroid carcinoma [152]. This proximity may be induced by genotoxic stress; for example, androgen stimulation coupled with the genotoxic stress of radiation was shown to generate gene fusions through “induced proximity” between *TMPRSS2* and *ERG* (located on chromosome 21q22.2, approximately 3 Mb apart) as well as between *TPMRSS2* and *ETV1* (located on chromosome 7) [153, 154] (Fig. 3a).

Another phenomenon, called chromothripsis, describes the frequent occurrence of massive chromosomal aberrations localized to only one or two chromosomes, with fragments of chromosome joined randomly [155, 156]. Chromothripsis may be responsible for the generation of numerous, apparently random passenger gene fusions that are retained in the multiclonal cells of epithelial cancers, as well as loss-of-function fusions involving tumor suppressors, likely involving the non-homologous end-joining DNA repair system (Fig. 3b).

Several cancer-causing viruses, such as Epstein–Barr virus (EBV), Kaposi's sarcoma-associated herpesvirus (KSHV), human papilloma virus (HPV), hepatitis B and C viruses (HBV and HCV), and Merkel cell polyomavirus (MCV), integrate into human genomic DNA at defined hotspots as well as seemingly randomly [157]. Viral integration events have been associated with chromosomal aberrations, such as *MYC* amplification in HPV-positive genital carcinoma [158], and not uncommonly, loss of gene function [159, 160] or gene fusions involving viral–human sequences have been reported [161, 162]. The recent report of a recurrent gene fusion of *UBR5* on 8q22.3 and *ZNF423* on 16q12.1 (*UBR5*-*ZNF423*) in 8 % of EBV-associated primary nasopharyngeal carcinomas suggests a driver function of this gene fusion in a subset of nasopharyngeal cancers [163].

## Additional files

Additional file 1:
**Recurrent gene fusions in epithelial cancers. **Summary of recurrent gene fusions in epithelial carcinoma across different tissues. **a** Gene fusions with common 5′ and 3′ genes. **b** Multiple 5′ partners with common 3′ genes. **c** Common 5′ gene partners with multiple 3′ genes. (DOCX 25 kb) Additional file 2:
**Clinical trials involving gene fusions in epithelial cancers.** (PDF 287 kb)

## Abbreviations

ACC: Adenoid cystic carcinoma
ALL: Acute lymphoblastic leukemia
AML: Acute myeloid leukemia
APL: Acute promyelocytic leukemia, cholangio cholangiocarcinoma
CML: Chronic myeloid leukemia
CRC: Colorectal carcinoma
CRPC: Castration-resistant prostate cancer
EBRT: External beam radiation therapy
EBV: Epstein–Barr virus
EST: Expressed sequence tag
FDA: Food and drug administration
FTC: Follicular thyroid carcinoma
GSI: γ-secretase inhibitor
HBV: Hepatitis B virus
HCV: Hepatitis C virus
HDR: High dose rate
HPV: Human papilloma virus
KSHV: Kaposi's sarcoma-associated herpesvirus
MASC: Mammary analog secretory carcinoma of salivary glands
MCV: Molluscum contagiosum virus
MEC: Mucoepidermoid carcinoma
MLL: Mixed lineage leukemia
MTC: Medullary thyroid cancer
nccRCC: non-clear-cell renal cell carcinoma
NGS: Next-generation sequencing
NICD: NOTCH intracellular domain
NMC: NUT midline carcinoma
NSCLC: Non-small-cell lung carcinoma
ORF: Open reading frame
Ph: Philadelphia chromosome
PLGA: Pediatric low grade astrocytoma
PTC: Papillary thyroid cancer
RACE 3′: Rapid amplification of cDNA ends
RCC: Renal cell carcinoma
RMC: Renal medullary carcinoma
TCGA: The Cancer Genome Atlas
TKI: Tyrosine kinase inhibitor
UTR: Untranslated region
