# Supplementary material for: Landscape of gene fusions in epithelial cancers: seq and ye shall find
Source: Genome Med. 2015 Dec 18;7:129. doi: 10.1186/s13073-015-0252-1 (PMC4683719; doi:10.1186/s13073-015-0252-1)
Supplement: Additional file 1: — Recurrent gene fusions in epithelial cancers. Summary of recurrent gene fusions in epithelial carcinoma across different tissues. a Gene fusions with common 5′ and 3′ genes. b Multiple 5′ partners with common 3′ genes. c Common 5′ gene partners with multiple 3′ genes. (DOCX 25 kb) [file 13073_2015_252_MOESM1_ESM.docx]

| **Additional File 1.** Recurrent gene fusions in epithelial cancers | | |
| --- | --- | --- |
| **(a) Gene fusions with common 5**′ **and 3**′ **genes** | | |
| **Tissues** | **5**′ **Gene** | **3**′ **Gene** |
| Intrathoracal, Bladder, Eye, Larynx, Lung, Nasal Cavity/Paranasal Sinuses, Nasopharynx, Skeleton, Thymus | *BRD4* | *NUT* |
| Intrathoracal | *BRD3* |  |
| Breast, Kidney, Large Intestine, Ovary, Stomach, Uterus Corpus | *CCND1* | *TACSTD2* |
| Salivary Gland | *EWSR1* | *POU5F1* |
| Skin |  |  |
| Lung, Nasopharynx, Oral Cavity, Salivary Gland, Skeleton, Skin, Thyroid, Uterus/Cervix | *CRTC1* | *MAML2* |
| Salivary Gland | *CRTC3* |  |
|  | *TORC1* |  |
| Large Intestine | *LACTB2* | *NCOA2* |
| Stomach | *CLDN18* | *ARHGAP26* |
| Stomach | *CD44* | *SLC1A2* |
| **(b) Multiple 5**′ **partners for a common 3**′ **gene** | | |
| **Tissues** | **5**′ **Gene** | **3**′ **Gene** |
| Breast, Large Intestine, Lung | *EML4* | *ALK* |
| Lung | *KIF5B* |  |
|  | *TFG* |  |
| Kidney | *VCL* |  |
| Stomach | *AGTRAP* | *BRAF* |
| Thyroid | *AKAP9* |  |
| Brain | *FAM131B* |  |
| Skin | *FCHSD1* |  |
| Brain | *KIAA1549* |  |
| Prostate | *SLC45A3* |  |
| Prostate | *ESRP1* | *RAF1* |
| Brain | *SRGAP3* |  |
| Salivary Gland | *HMGA2* | *NFIB* |
| Breast, Nasopharynx, Skin | *MYB* |  |
| Thyroid | *TFG* | *NTRK1* |
|  | *TPM3* |  |
|  | *TPR* |  |
| Breast, Salivary Gland | *ETV6* | *NTRK3* |
| Breast | *RIF1* | *PKD1L1* |
| Ovary | *TNS3* |  |
| Thyroid | *CCDC6* | *RET* |
|  | *ERC1* |  |
|  | *GOLGA5* |  |
|  | *HOOK3* |  |
|  | *KTN1* |  |
|  | *NCOA4* |  |
|  | *PCM1* |  |
|  | *PRKAR1A* |  |
|  | *RFG9* |  |
|  | *TRIM24* |  |
|  | *TRIM27* |  |
|  | *TRIM33* |  |
| Lung | *KIF5B* |  |
| Brain | *GOPC* | *ROS1* |
| Lung | *CD74* |  |
|  | *SLC34A2* |  |
| Prostate | *FKBP5* | *ERG* |
|  | *HERPUD1* |  |
|  | *NDRG1* |  |
|  | *SLC45A3* |  |
|  | *TMPRSS2* |  |
|  | *ACSL3* | *ETV1* |
|  | *AX747630* |  |
|  | *C15ORF21* |  |
|  | *EST14* |  |
|  | *FLJ35294* |  |
|  | *FOXP1* |  |
|  | *HERVK17* |  |
|  | *HERVK22Q11* |  |
|  | *HNRNPA2B1* |  |
|  | *KLK2* |  |
|  | *SLC45A3* |  |
|  | *TMPRSS2* |  |
|  | *CANT1* | *ETV4* |
|  | *DDX5* |  |
|  | *KLK2* |  |
|  | *TMPRSS2* |  |
|  | *SLC45A3* | *ETV5* |
|  | *TMPRSS2* |  |
|  | *SLC45A3* | *FLI1* |
|  | *TMPRSS2* | *SKIL* |
|  | *SLC45A3* |  |
|  | *MIPEP* |  |
|  | *PIPOL1* |  |
|  | *ACPP* |  |
|  | *HMGN2P46* |  |
| Breast | *NFIX* | *MAST1* |
|  | *ZNF700* |  |
|  | *TADA2A* |  |
|  | *ARID1A* | *MAST2* |
|  | *GPBP1L1* |  |
|  | *SEC16A* | *NOTCH1* |
|  | *SEC22B* | *NOTCH2* |
|  | *BCAS4* | *BCAS3* |
|  | *ERBB2* |  |
|  | *MED13* |  |
|  | *CDC6* | *IKZF3* |
|  | *MED1* |  |
|  | *VAPB* |  |
|  | *BC017255* | *VMP1* |
|  | *CLTC* |  |
|  | *INTS2* |  |
|  | *RPS6KB1* |  |
|  | *TRIM37* |  |
| Salivary Gland | *CHCHD7* | *PLAG1* |
|  | *CTNNB1* |  |
|  | *FGFR1* |  |
|  | *LIFR* |  |
|  | *TCEA1* |  |
| Kidney | *ASPSCR1* | *TFE3* |
|  | *CLTC* |  |
|  | *NONO* |  |
|  | *PRCC* |  |
|  | *SFPQ* |  |
| Colorectal, Prostate | *EIF3E* | *RSPO2* |
|  | *PTPRK* |  |
|  | *GRHL2* |  |
| **(c) Common 5**′ **gene partners with multiple 3**′ **genes** | | |
| **Tissues** | **5**′ **Gene** | **3**′ **Gene** |
| Ovary | *BCAS3* | *ARHGAP15* |
| Breast |  | *HOXB9* |
|  |  | *MED13* |
|  |  | *TG* |
| Kidney | *CLTC* | *TFE3* |
| Breast |  | *VMP1* |
| Breast | *ERBB2* | *BCAS3* |
| Ovary |  | *PGAP3* |
| Breast | *ETV6* | *ITPR2* |
|  |  | *NTRK3* |
| Salivary Gland |  |  |
| Salivary Gland | *EWSR1* | *ATF1* |
| Pancreas |  | *FLI1* |
| Breast | *PAPOLA* | *AK7* |
| Ovary |  | *HIP1R* |
| Lung | *TFG* | *ALK* |
| Thyroid |  | *NTRK1* |
| Thyroid | *TPM3* | *NTRK1* |
|  |  | *TPR* |
