# Supplementary material for: Landscape of gene fusions in epithelial cancers: seq and ye shall find
Source: Genome Med. 2015 Dec 18;7:129. doi: 10.1186/s13073-015-0252-1 (PMC4683719; doi:10.1186/s13073-015-0252-1)
Supplement: Additional file 2: — Clinical trials involving gene fusions in epithelial cancers. (PDF 287 kb) [file 13073_2015_252_MOESM2_ESM.pdf]

Additional file 2. Clinical trials involving gene fusions in epithelial cancers

Gene fusion(s)	ClinicalTrials.gov ID	Title	Condition	Intervention
ALK	NCT00932451/ NCT00932893	An Investigational Drug, PF-02341066, Is Being Studied In Patients With Advanced Non-Small Cell Lung Cancer With A Specific Gene Profile Involving The Anaplastic Lymphoma Kinase (ALK) Gene	NSCLC	PF-02341066
ALK	NCT01100840	A Retrospective Study of Biomarkers in Non-Small Cell Lung Cancer	NSCLC	
ALK	NCT01154140	A Clinical Trial Testing The Efficacy Of Crizotinib Versus Standard Chemotherapy Pemetrexed Plus Cisplatin Or Carboplatin In Patients With ALK Positive Non Squamous Cancer Of The Lung	Non-squamous lung cancer	Treatment
ALK	NCT01449461	A Phase 1/2 Study of the Oral ALK/EGFR Inhibitor AP26113	Advanced malignancies; NSCLC	AP26113
ALK	NCT01562015	A Study of Ganetespib in Subjects With ALK-Positive Non-Small-Cell Lung Cancer (NSCLC)	NSCLC	Ganetespib
ALK	NCT01994057	A Retrospective Pharmacogenomics Research of EGFR-TKIs, Gefitinib and Erlotinib, in Non-small Cell Lung Cancer (NSCLC)Treatment	NSCLC; EGFR-TKI-resistant mutations; EGFR-TKI-sensitizing mutations; germline mutations	
ALK	NCT02010047	Comparison of quantitative PCR to IHC and FISH for Detection of ALK Fusion Mutations in FFPE Tissue From NSCLC Patients	NSCLC	ALK quantitative PCR assay
ALK	NCT02041468/ NCT02228421	Study to Evaluate Resistance Mechanisms and Real-world Pharmacoeconomics of Crizotinib in NSCLC Patients	Metastatic NSCLC	
ALK	NCT02289144	Ceritinib (LDK378) in Mutation and Oncogene Directed Therapy in Metastatic or Locally Advanced Anaplastic/Undifferentiated Thyroid Cancer	Locally advanced metastatic or anaplastic/undifferentiated thyroid cancer; anaplastic/undifferentiated thyroid cancer	Ceritinib (LDK378)
ALK/ *BRD-NUT*	NCT02259114	A Phase IB Trial With OTX015, a Small Molecule Inhibitor of the Bromodomain and Extra-Terminal (BET) Proteins, in Patients With Selected Advanced Solid Tumors	NUT midline carcinoma; triple-negative breast cancer; NSCLC with rearranged ALK gene/fusion protein or	OTX015

			KRAS mutation; CRPC; pancreatic ductal adenocarcinoma	
ALK/RET	NCT02314481	Deciphering Antitumour Response and Resistance With Intratumour Heterogeneity	NSCLC	MPDL3280A, vemurafenib, alectinib, trastuzumab, emtansine
EML4-ALK	NCT01662635	Clinicopathological Features of NSCLC Patients Associated With the Chromosome 2p (EML4-ALK)	NSCLC	
TPM4-ALK	NCT01700582	French National Observatory of the Patients With Non-small Cell Lung (NSCLC) and Molecular Testings	NSCLC; TPM4–ALK fusion protein expression; *KRAS* gene mutation; *BRAF* gene mutation	
KIAA1549-BRAF	NCT01089101	Selumetinib in Treating Young Patients With Recurrent or Refractory Low Grade Glioma	Childhood cerebellar astrocytoma; childhood mixed glioma; childhood pilocytic astrocytoma; neurofibromatosis type 1; recurrent childhood anaplastic oligodendroglioma; recurrent childhood brain stem glioma; recurrent childhood cerebellar astrocytoma; recurrent childhood oligodendroglioma; recurrent childhood pilocytic astrocytoma; recurrent childhood visual pathway glioma	Laboratory biomarker analysis, pharmacological study, selumetinib
KIAA1549-BRAF	NCT02285439	Phase I/II Study of MEK162 for Children With Ras/Raf Pathway Activated Tumors	Low-grade gliomas; malignant brain neoplasms; brain; soft tissue neoplasms	MEK162
ETS	NCT00480090	A Phase II Study of Ara-C (Cytarabine) in Men With Androgen	Prostate cancer	Ara-C (cytarabine)

		Independent Prostate Cancer		
ETS	NCT01444820	Hypofractionated, Dose Escalation Radiotherapy for High Risk Adenocarcinoma of the Prostate	Prostate cancer	Hypofractionation or conventional radiation
ETS	NCT01576172	Abiraterone Acetate and Prednisone With or Without Veliparib in Treating Patients With Metastatic Hormone-Resistant Prostate Cancer	Hormone-resistant prostate cancer; recurrent prostate carcinoma; stage IV prostate cancer	Abiraterone acetate, laboratory biomarker analysis, prednisone, veliparib
ETS	NCT01858441	Pharmacogenetic Study in Castration-resistant Prostate Cancer Patients Treated With Abiraterone Acetate	CRPC	Abiraterone, acetate
ETS	NCT02288936	Analyze the Predictive Value of Gene TMPRSS2-ETS in Response to Enzalutamide in Patients With Prostate Cancer	Hormone-refractory prostate cancer	Enzalutamide
ETS	NCT02303327	Comparative Study of Radiotherapy Treatments to Treat High Risk Prostate Cancer Patients	Prostate cancer	EBRT plus HDR brachytherapy boost, hypofractionated dose escalation radiotherapy, androgen deprivation therapy
FGFRx	NCT02150967	A Phase II, Single Arm Study of BGJ398 in Patients With Advanced Cholangiocarcinoma	Advanced cholangiocarcinoma	BGJ398
FGFRx	NCT02265341	Ponatinib Hydrochloride in Treating Patients With Advanced Biliary Cancer With FGFR2 Fusions	Malignant hepatobiliary neoplasm	Ponatinib hydrochloride, laboratory biomarker analysis, questionnaire administration, quality-of-life assessment
HER2-GRB7	NCT01956253	Single Subject Neratinib in Bladder Cancer	Bladder cancer	Neratinib
LMNA-NTRK1	NCT02122913	Oral TRK Inhibitor LOXO-101 for Treatment of Advanced Adult Solid	Metastatic soft tissue sarcoma (solid	LOXO-101

		Tumors	tumors)	
MET, RET, AXL, NTRK1, or *NTRK3*	NCT02219711	Phase 1/1b Study of MGCD516 in Patients With Advanced Cancer	Advanced cancer	MGCD516
PAX8-PPARγ	NCT01655719	Pioglitazone in Follicular or Follicular Variant of Papillary Thyroid Cancers	Follicular thyroid carcinoma; follicular variant of papillary thyroid carcinoma	Pioglitazone
RET	NCT01639508	Cabozantinib in Patients With RET Fusion-Positive Advanced Non-Small Cell Lung Cancer and Those With Other Genotypes: ROS1 or NTRK Fusions or Increased MET or AXL Activity	NSCLC	Cabozantinib
RET	NCT01813734	Ponatinib in Advanced NSCLC with RET Translocations	NSCLC	Ponatinib
RET	NCT01823068	Vandetanib in Advanced NSCLC With RET Rearrangement	NSCLC	Vandetanib
RET, RAF, PDGFR	NCT02013089	A Pilot Study of Genomic Sequencing Guided Individualized Therapy in Gastrointestinal Cancers, GITIC Study	Gastrointestinal cancers	Erlotinib or gefitinib, everolimus, imatinib, sorafenib or sunitinib, vandetanib
ROS	NCT01596374	Clinical Relevance of ROS (V-ros UR2 Sarcoma Virus Oncogene Homolog) Aberrations in Solid Tumours	Solid cancers	
RSPO	NCT02278133	Study of WNT974 in Combination With LGX818 and Cetuximab in Patients With BRAF-mutant Metastatic Colorectal Cancer (mCRC) and Wnt Pathway Mutations	Metastatic colorectal cancer	WNT974, LGX818, cetuximab

CRPC castration-resistant prostate cancer, *EBRT* external beam radiation therapy, *HDR* high dose rate, *NSCLC* non-small-cell lung cancer, *TKI* tyrosine kinase inhibitor
